# Longitudinal Trajectory of Cognition, Brain Morphometry, and Brain Predicted Age in Unaffected First‐Degree Relatives of Patients With Bipolar Disorder

**DOI:** 10.1111/bdi.70050

**Published:** 2025-08-13

**Authors:** Hanne Lie Kjærstad, Julian Macoveanu, Alexander Tobias Ysbæk‐Nielsen, Viktoria Damgaard, Sophia Frangou, Gitte M. Knudsen, Klara Coello, Sharleny Stanislaus, Maria Faurholt‐Jepsen, Maj Vinberg, Lars Vedel Kessing, Kamilla Woznica Miskowiak

**Affiliations:** ^1^ Neurocognition and Emotion in Affective Disorders (NEAD) Centre, Psychiatric Centre Copenhagen, Mental Health Services, Capital Region of Denmark and Department of Psychology, University of Copenhagen Copenhagen Denmark; ^2^ Copenhagen Affective Disorder Research Centre (CADIC), Psychiatric Centre Copenhagen, Frederiksberg Hospital, Mental Health Services, Capital Region of Denmark Frederiksberg Denmark; ^3^ Department of Psychology University of Copenhagen Copenhagen Denmark; ^4^ Department of Psychiatry, Icahn School of Medicine at Mount Sinai New York USA; ^5^ Department of Psychiatry University of British Columbia Vancouver Canada; ^6^ Neurobiology Research Unit, Copenhagen University Hospital, Rigshospitalet Copenhagen Denmark; ^7^ Department of Clinical Medicine, Faculty of Health and Medical Sciences University of Copenhagen Copenhagen Denmark; ^8^ Mental Health Centre, Northern Zealand, Copenhagen University Hospital – Mental Health Services CPH Copenhagen Denmark

**Keywords:** amygdala, bipolar disorder, brain age, endophenotype, MRI, unaffected relatives

## Abstract

**Introduction:**

Prior research suggests structural brain abnormalities and cognitive difficulties in patients with bipolar disorder. Although there is some evidence that similar structural and cognitive changes may also be present in unaffected relatives (UR) of patients with bipolar disorder, it is not known whether they remain static or aggravate over time. In this study, we investigate the longitudinal trajectories of cognition and brain structure in UR.

**Methods:**

Longitudinal neurocognitive and MRI data were acquired at baseline from UR (*n* = 72) and healthy controls (HC; *n* = 65) and at 15 (±4) months follow‐up (UR *n* = 32; HC *n* = 38). The differential trajectories between UR and HC in neurocognitive performance, white matter volume, regional cortical gray matter (GM) volume and thickness, hippocampal and amygdala volumes, and the difference between biological age and age estimated from brain MRI (brainPAD) were investigated using linear mixed models.

**Results:**

UR showed subtle impairments in processing speed, which normalized at follow‐up to levels comparable to HC. At both time points, UR showed stable enlargement of amygdalae compared to HC. There was a significant group‐by‐time interaction effect for the GM volume in the left superior temporal gyrus, driven by UR at baseline displaying larger GM volume compared to HC, which normalized over time. There was no significant difference between UR and HC in brainPAD.

**Conclusion:**

Bilaterally enlarged amygdala and larger temporal GM volume in UR compared to HC may reflect a vulnerability factor for bipolar disorder. Longer follow‐up times are needed to elucidate structural predictors of risk of subsequent illness onset.

## 1 Introduction

Bipolar disorder (BD) is a chronic and recurrent mood disorder marked by severe mood fluctuations and impaired psychological functioning [1]. It is highly heritable, with twin studies showing a genetic risk of 60%–85% [2, 3]. Whereas the population incidence of BD is 1%–4%, the cumulative incidence of BD increases to 10%–25% in people with one affected first‐degree relative and to 10%–50% in offspring of two parents with BD [4, 5]. Neuroimaging studies of structural brain changes in BD have demonstrated widespread white matter (WM) abnormalities [6] and aberrant frontal and temporal lobe gray matter (GM) [7, 8], which are associated with cognitive difficulties [9, 10]. Investigations of longitudinal structural changes in BD have shown GM volume loss in prefrontal and anterior cingulate cortex (ACC) over time [11, 12]. Patients with BD also exhibit reduced cortical thickness in frontotemporal regions, which show accelerated thinning over time [13, 14], although results are somewhat conflicting [15].

Interpretation of these structural and cognitive abnormalities in BD is challenging as they may reflect disease expression, consequences of medication, or vulnerability‐related mechanisms [16]. Examination of unaffected relatives (UR) offers a unique opportunity to distinguish vulnerability mechanisms without confounding clinical factors. Most *cross‐sectional* studies examining UR of patients with BD report structural changes similar to their affected probands, including regional reductions in WM integrity, aberrant cortical thickness, and GM volumes in frontotemporal and sub‐cortical regions [17, 18, 19, 20]. Although studies typically show abnormal amygdala and hippocampus volume in the early course of BD, evidence for such abnormalities in UR is lacking, suggesting that these abnormalities are related to other clinical factors such as disease expression [21]. Longitudinal evidence suggests accelerated cortical thinning in lateralized frontal regions in unaffected youth [22] as well as in UR who remained well [23] during a 2‐year follow‐up time compared to healthy controls (HC). UR who developed major depressive disorder during the 2‐year follow‐up showed thickening of the left inferior frontal gyrus and left precentral gyrus relative to UR who remained well [23]. UR also exhibited greater frontotemporal GM volume compared to HC, which reduced over time in one study [22], but remained stable in another study [24]. In contrast, no significant trajectory differences between UR and HC have been found in subcortical volumes or WM integrity [25, 26].

An influential hypothesis is that brain, and possibly cognitive abnormalities, in BD may reflect deviations from the normative pattern of age‐related brain changes. Such investigations have been made possible through the use of machine learning techniques that can predict age from structural neuroimaging data. The brain‐predicted‐age‐difference (brainPAD) is the difference between neuroimaging predicted and chronological age and represents an individualized estimate of brain aging [27]. Higher brainPAD, suggesting accelerated aging, is observed in patients with established and chronic BD; albeit very subtle, averaging about 1–2 years [28, 29, 30]. Patients in the early stages of BD and UR exhibit comparable brainPAD to HC [31, 32], with UR showing a deceleration over a two‐year follow‐up time, suggesting a maturational lag [32]. The findings from these studies may point toward accelerated aging being a part of the disease process rather than a marker of vulnerability.

This study addresses critical gaps in the longitudinal evolution of brain structural and cognitive changes and brainPAD in unaffected first‐degree relatives of patients with BD compared to HC over an average 15‐month follow‐up period. Based on previous longitudinal studies in UR, e.g., [22, 23], we hypothesized that UR and HC would exhibit differential trajectory changes in GM volume and cortical thickness in prefrontal and temporo‐limbic regions. Further, given the lack of trajectory differences found in previous studies, e.g., [25, 26, 32], we expected no change in WM trajectory, brainPAD, or cognition.

## 2 Methods

### 2.1 Study Design and Participants

The current study is a longitudinal investigation of unaffected first‐degree relatives of patients with BD and HC from the ongoing Bipolar Illness Onset (BIO) study [33]. The sample of UR comprised siblings (*n* = 67) and offspring (*n* = 5) of patients with newly diagnosed BD referred to the Copenhagen Affective Disorder Clinic, which offers treatment for newly diagnosed BD for the 1.8 million people in the catchment area of the Capital Region of Denmark [33]. Six of the UR had one other genetically related UR sibling in the study. Relatives were contacted upon consent from their affected proband and were included if they met inclusion criteria, i.e., age 15–40 years and having no personal lifetime history of mental disorders. Age‐ and sex‐matched unrelated individuals were recruited from the University Hospital, Rigshospitalet, Blood Bank, as HC who had no personal or family (up to first‐degree relatives) history of mental disorders. Upon study inclusion, the absence of a personal history of psychiatric illness in all participants was ascertained by a semi‐structured interview with the Schedules for Clinical Assessment in Neuropsychiatry (SCAN) [34] by PhD students in Medicine or Psychology. Mood symptoms were assessed with the Hamilton Depression Rating Scale (HDRS‐17) [35] and the Young Mania Rating Scale (YMRS) [36]. Exclusion criteria for all participants included an HDRS or YMRS total score > 14, a history of severe brain injury, a neurological disorder (including dementia), current severe somatic illness, and/or current substance abuse disorder. All included participants were fluent in Danish.

The authors of this paper declare that all procedures contributing to this work comply with the ethical standards of the relevant national and institutional committees on human experimentation and with the Helsinki Declaration of 1975, as revised in 2008. The study was approved by the Committee on Health Research Ethics of the Capital Region of Denmark (protocol number: H‐7‐2014‐007) and the Danish Data Protection Agency, Capital Region of Copenhagen (protocol number: RHP‐2015‐023). Informed consent was obtained from all participants prior to study participation.

### 2.2 Procedure

Participants attended an initial screening visit conducted by a PhD student in Medicine or Psychology, in which data regarding demographic information (age, sex, years of education), clinical characteristics, psychiatric history, and diagnostic interview were collected. Eligible participants were invited to two separate testing sessions, approximately 15 months apart. The assessments, instruments, and MRI scanner acquisition and sequences used were the same for all participants at both baseline and follow‐up, consisting of (i) mood assessments (HDRS‐17 and YMRS); (ii) assessments of predicted full‐scale intelligence quotient (IQ) and neurocognitive testing; (iii) functioning [37] and quality of life [38]; and (iv) T1‐weighted structural MRI. The Danish version of the National Adult Reading Test (DART) was only administered at baseline. Due to limited financial resources, about half of the original baseline sample was randomly invited to the follow‐up assessment.

### 2.3 Measures

#### 2.3.1 Functioning and Quality of Life

Participants' functioning in six domains (interpersonal relationships, autonomy, occupational, financial, subjective cognitive functioning, leisure time) was assessed with the Functional Assessment Short Test (FAST) [37] [39]. Quality of life was assessed with the European Quality of Life 5‐Domain Questionnaire EQ‐5D [38] to generate index scores.

#### 2.3.2 Neurocognition

Predicted full‐scale IQ was calculated from the DART. Neurocognitive performance was further assessed with a comprehensive test battery including: Coding and Digit Span Forward from the Repeatable Battery for the Assessment of Neuropsychological Status (RBANS) [40], Letter‐Number Sequencing from Wechsler's Adult Intelligence Scale 3rd edition (WAIS‐III) [41], the Trail Making Test‐A and B (TMT‐A/TMT‐B) [42], the Rey Auditory Verbal Learning Test (RAVLT) [43], verbal fluency S and D [44], and the Spatial Working Memory (SWM) test and the Rapid Visual Information Processing (RVP) test from the Cambridge Neuropsychological Test Automated Battery (CANTAB). Different versions of the RAVLT (list AB, GeAB, and Cr‐AB) and RBANS coding (version A and B) were counterbalanced at baseline and follow‐up to reduce the risk of practice effects. Raw scores from the neurocognitive tests were transformed to z‐Scores based on the HC means and standard deviations at baseline. z‐Scores for TMT‐A, TMT‐B, RVP latency, SWM between errors, and SWM strategy were inverted to higher z‐Scores consistently reflecting better performance. z‐Scores were averaged into four cognitive domains: processing speed (RBANS Coding, TMT‐A); sustained attention (RVP A' and mean latency); verbal learning and memory (RAVLT Trial 1–5 correct, Trial 6 correct, Delayed recall and Recognition); working memory and executive functions (TMT‐B, SWM strategy and between errors, Letter‐number sequencing, Digit span, and Verbal Fluency letters “S” and “D”). A global composite score was calculated by averaging the *z*‐Scores of the four cognitive domains.

### 2.4 Structural MRI Data Processing and Statistical Analyses

#### 2.4.1 MRI Acquisition Protocol

Magnetic resonance imaging data were acquired at Copenhagen University Hospital, Rigshospitalet using a 3 Tesla Siemens Prisma Scanner with a 64‐channel head–neck coil. T1‐weighted structural images were acquired using a 3D MPRAGE sequence and the following parameters: FOV = 230 x 230 mm, slice thickness = 0.9 mm, TR = 1900 ms, TE = 2.58 ms, flip angle = 9°, distance factor = 50%. All scans were corrected for B0 field geometric distortions and visually inspected to rule out overt structural brain abnormalities or artifacts.

#### 2.4.2 MRI Data Processing

Cortical reconstruction and volumetric segmentation were performed using the FreeSurfer image analysis suite v7.1.0 (http://surfer.nmr.mgh.harvard.edu/). Individual B_0_ field distortion‐corrected T1‐weighted images were processed with the longitudinal FreeSurfer stream in three steps [45]. Each individual scan was first processed according to standard procedure [46, 47], which included correction for intensity non‐uniformity, intensity normalization, skull strip, automatic parcellation of cortical regions based on the Desikan‐Killany Atlas [48] and segmentation of subcortical brain regions (including the amygdalae and the hippocampi) based on Fischl, Salat, Busa, Albert, Dieterich, Haselgrove, Van Der Kouwe, Killiany, Kennedy, and Klaveness [47]. Thereafter, regional measures of cortical thickness and surface area and regional subcortical volumes were computed together with global measures of cortical gray and WM volumes and the total intracranial volume (TIV). In a second step, an unbiased within‐subject template space and image was created using robust, inverse consistent registration. In a final processing step, each individual MRI scan was processed again using the subject template information for improved accuracy of the estimated anatomical measurements. Only the processing output of the final stage was used for statistical analysis. Quality assessment of the cortical reconstruction relied on the Cortical Quality Control Protocol 2.0 [49] by the Enhancing NeuroImaging Genetics through Meta‐Analysis (ENIGMA) Consortium to detect errors, which we subsequently corrected manually.

#### 2.4.3 Statistical Analysis of the Structural MRI Data

*Vertex‐wise cortical analysis.* We assessed group differences in cortical thickness and volume using linear mixed effect (LME) models implemented in MATLAB (Mathworks Inc. Natick, Massachusetts) version R2021a [50]. Vertex thickness or volume was entered in an LME model with a random intercept, familial relationship as a random factor, and group (UR and HC), group*follow‐up time as fixed effects as well as age, sex, and TIV to account for these variables. To test our hypothesis regarding abnormalities in UR in temporal cortical thickness and volume, we constructed bilateral temporal cortex masks by adding the superior, middle, and inferior temporal gyri and the temporal pole from the automated cortical parcellation according to the Desikan‐Killiany Atlas [48]. We assessed significant clusters at an alpha level of 0.05 after correction of the *p*‐value threshold for multiple comparisons using the false discovery rate (FDR) method across vertexes within the constructed temporal mask or across the entire cortex for the exploratory cortex analysis.

*Cerebral white matter, amygdala, and hippocampal volume analyses*. To assess differences in total cerebral WM, amygdala, and hippocampus volume trajectories in UR compared to HC, we constructed separate LME models with time (baseline, follow‐up), group (UR, HC), and group‐by‐time interaction as fixed factors, familial relationship as a random factor, and adjusting for sex, age, time between MRI sessions, and TIV. We used the default alpha level of significance of *p* < 0.05 (two‐tailed).

#### 2.4.4 Computation of BrainPAD

We computed brainPAD in all participants using the pyment software package for an accurate brain age prediction using a deep convolutional neural network model [51]. We calculated participants' brainPAD by subtracting chronological age from brain‐predicted age [32]. To assess differences in brainPAD trajectories between groups, we implemented a similar LME in SPSS as above, adjusting for age and sex since the brain age prediction algorithm is dependent on these factors.

### 2.5 Statistical Analyses of Clinical, Demographic, and Neurocognitive Data

Statistical analyses were performed using the Statistical Package for the Social Sciences (SPSS) version 25 (IBM Corporation, Armonk, NY). Data normality distribution was explored using the Shapiro–Wilk test. To assess differences in baseline demographic, clinical, and cognitive characteristics between UR and HC, we employed independent samples *t*‐test, nonparametric Mann–Whitney U test, and Pearson's chi‐square (χ^2^). To assess differences between UR and HC over time, linear mixed model analyses were performed with time (baseline, follow‐up), group (UR, HC) and time‐by‐group interaction as fixed factors, familial relationship as a random factor, adjusting for IQ and time between assessments. *p*‐values were FDR corrected for multiple comparisons using the Benjamini–Hochberg procedure.

We conducted exploratory post hoc Pearson correlation analyses to explore associations between baseline aberrant brain structure or neurocognition with subsyndromal depression and mania symptoms and level of functioning. The correlation analysis was performed only in the UR group to avoid inflating the effects [52]; *p*‐values were FDR‐adjusted.

## 3 Results

### 3.1 Participants

Baseline structural MRI data were collected from 140 participants, including 74 UR and 66 HC. Follow‐up structural MRI was collected an average of 15 (±4) months later for 32 UR and 38 HC. Three participants (UR *n* = 2 and HC *n* = 1) developed Major Depressive Disorder between baseline and follow‐up scans and were therefore excluded from all analyses. Both UR and HC were free of psychiatric illness and were not taking psychotropic medication. Most of the MRI scans and neuropsychological testing (98%) were conducted 0–3 days apart (same day = 74%; 1 day = 11%; 2 days = 10%; and 3 days = 3%).

### 3.2 Baseline Demographic and Clinical Characteristics

In terms of age, sex, years of education, subsyndromal mania symptoms, and quality of life, UR and HC were similar (*p*‐values ≥ 0.10). Both HC and UR had high predicted IQ scores, which were marginally lower in UR (*p* = 0.01) and UR reported more functional difficulties than HC (*p* = 0.001), specifically within the domains of cognitive functioning, financial issues, and leisure time (p‐values ≤ 0.04) (Table 1). Participants with only baseline assessments were comparable to those with both assessments, suggesting an absence of bias in the follow‐up sample (Tables S1 and S2).

**TABLE 1:** Demographic and clinical characteristics of unaffected relatives of patients with bipolar disorder and healthy controls at baseline and 15‐month follow‐up.

	Baseline				Follow‐up				Linear mixed models time‐by‐group interaction	
	Unaffected relatives	Healthy controls	*t*/*U*/*χ* ^2^	*p*	Unaffected relatives	Healthy controls	*t*/*U*	*p*	*f*	*p*
*N*	72	65			32	38				
Sex, *n* (% female)	36 (50%)	40 (62%)	1.84	0.18						
Age	27.39 (7.18)	29.55 (9.62)	2181.00	0.49						
Predicted full‐scale IQ	110.29 (6.77)	112.74 (4.76)	1634.50	0.01						
Years of education	14.99 (2.90)	15.72 (2.27)	1.65	0.10	15.94 (3.01)	15.57 (1.92)	590.50	0.84	2.62	0.11
HDRS, total score	1.52 (2.20)	1.02 (1.17)	2176.50	0.54	1.88 (3.13)	0.95 (1.29)	566.50	0.60	0.44	0.51
YMRS, total score	0.80 (1.56)	0.77 (1.73)	2208.50	0.58	0.47 (0.76)	0.68 (1.28)	594.00	0.84	3.34	0.07
FAST, total score	3.96 (6.85)	1.17 (1.67)	1604.50	0.001	2.86 (3.48)	2.08 (4.55)	439.00	0.13	1.27	0.26
Autonomy	0.39 (1.09)	0.09 (0.29)	2074.00	0.09	0.45 (0.91)	0.11 (0.39)	458.00	0.06	1.42	0.24
Occupational functioning	0.85 (2.74)	0.12 (0.33)	2137.00	0.24	0.52 (1.70)	0.87 (3.40)	501.50	0.31	1.07	0.31
Cognitive functioning	1.04 (1.74)	0.48 (0.87)	1811.50	0.02	0.66 (1.08)	0.50 (0.95)	504.00	0.49	0.47	0.50
Financial issues	0.25 (0.65)	0.06 (0.30)	2052.50	0.04	0.21 (0.56)	0.08 (0.36)	504.00	0.23	0.17	0.68
Interpersonal relationships	0.72 (1.53)	0.26 (0.57)	2010.50	0.09	0.41 (0.78)	0.32 (0.66)	524.50	0.66	0.11	0.75
Leisure time	0.70 (1.20)	0.15 (0.40)	1702.50	< 0.001	0.62 (1.21)	0.21 (0.74)	438.50	0.04	1.63	0.21
Quality of life, EQ‐5D index score	0.96 (0.07)	0.97 (0.06)	1894.50	0.30	0.97 (0.08)	0.97 (0.06)	568.50	0.98	0	0.97
Discordant time, years ^a^	1.21 (1.29)									

Abbreviations: EQ‐5D = European Quality of Life—5 Dimensions; FAST = Functioning Assessment Short Test; HDRS = Hamilton Depression Rating Scale; YMRS = Young Mania Rating Scale.

^a^ Discordant time calculated as the time between affected proband's date of BD diagnosis and date of MRI assessment.

### 3.3 Change in Neurocognitive Functioning Over Time

**FIGURE 1:**
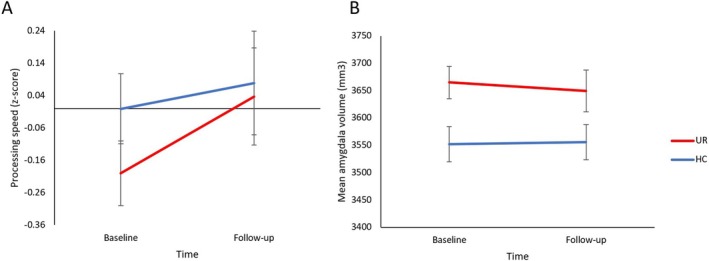
Unaffected relatives (UR) of patients with bipolar disorder demonstrated (A) significant improvement in processing speed (*p* = 0.004) compared to healthy controls (HC) whose performance remained stable over the 15‐month follow‐up time (*p* = 0.66) and (B) significantly greater amygdala volume compared to HC across both time points (*p* = 0.03).

At baseline, UR generally underperformed cognitively slightly compared to HC (absolute *z*‐score 0.12 to −0.26); which reached statistical significance at *p* < 0.05 for processing speed and working memory/executive function. These significant *p*‐values did, however, not reach statistical significance after adjusting for multiple comparisons (*p*‐values ≥ 0.1). At follow‐up, we saw no group differences even at uncorrected *p*‐values. There was a time‐by‐group interaction for processing speed (*F* (1, 78.60) = 8.18, *p* = 0.005, FDR corrected *p* = 0.03), driven by UR showing significant improvement over time (*p* = 0.004), whereas HC remained stable (*p* = 0.66) (Table 2; Figure 1A). Analyses of global cognition or other cognitive domains revealed no significant group‐by‐time interactions (ps ≥ 0.14) or main effects of group (ps ≥ 0.34). Neurocognitive performance in participants with only baseline assessment was comparable to those with both assessments (Tables S1 and S2).

**TABLE 2:** Neurocognitive performance for unaffected relatives of patients with bipolar disorder and healthy controls at baseline and 15‐month follow‐up.

	Baseline				Follow‐up				Linear mixed models time‐by‐group interaction	
	Unaffected relatives	Healthy controls	*t*/*U*	*p*	Unaffected relatives	Healthy controls	*t*/*U*	*p*	*f*	Uncorrected *P*‐value
Global cognition	−0.14 (0.45)	0.00 (0.56)	1.57	0.12	0.12 (0.51)	0.18 (0.60)	0.49	0.63	0.75	0.39
Processing speed	−0.26 (0.64)	0.00 (0.73)	2.10	0.04	0.09 (0.59)	0.02 (0.76)	0.43	0.67	8.18	0.005*
Sustained attention	0.12 (0.75)	0.00 (0.88)	0.88	0.38	0.51 (0.65)	0.53 (0.64)	0.14	0.89	1.19	0.28
Verbal learning and memory	−0.20 (0.98)	0.00 (0.86)	2067.50	0.24	−0.08 (0.96)	0.16 (0.81)	527.00	0.34	0.03	0.87
Working memory and executive function	−0.22 (0.49)	0.00 (0.61)	2.35	0.02	−0.05 (0.61)	0.01 (0.60)	0.44	0.66	2.19	0.14

* FDR corrected *p*‐value = 0.025.

### 3.4 Subcortical and Total WM Volume Analysis

UR showed larger amygdala volume than HC across both timepoints (F (1,121.15) = 4.96, *p* = 0.03), with no significant trajectory difference between the groups (*p* = 0.46) (Figure 1B). Analyses of total cerebral WM and hippocampal volume showed no significant difference between groups (ps ≥ 0.25).

### 3.5 Cortical Surface Analysis

**FIGURE 2:**
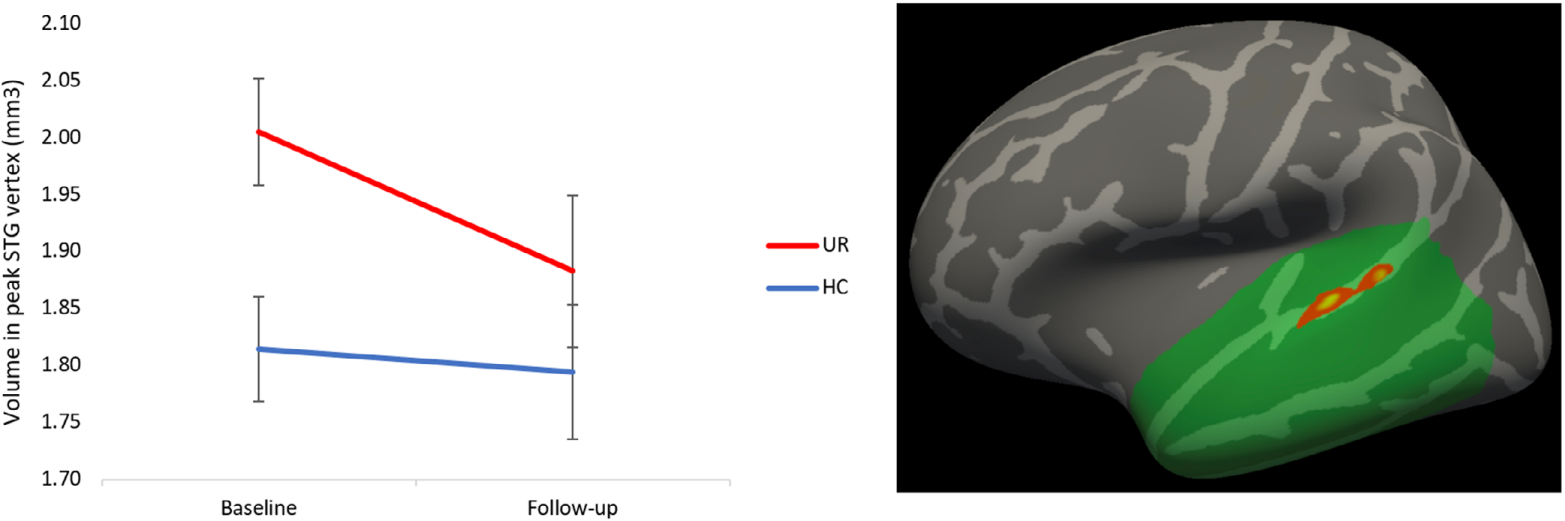
Unaffected relatives (UR) of patients with bipolar disorder had significantly larger left superior temporal gyrus (STG) volumes at baseline (*p* = 0.006) compared to healthy controls (HC). At follow‐up, however, a significant group‐by‐time interaction (*p* = 0.034) had evened out this difference.

There was a significant group‐by‐time interaction effect in the temporal cortex VOI (peak effect in the left superior temporal gyrus (STG) MNI x, y, z = −62, −23, 3; *p* < 0.001) (F (1, 67.32) = 21.53, *p* < 0.001), driven by UR showing a larger GM volume compared to HC across both timepoints (group effect F (1, 85.216) = 7.88, *p* = 0.006) but also a relative decrease over time compared to a longitudinally stable HC volume in this region (group‐by‐time F (1, 53.607) = 4.75, *p* = 0.03) (Figure 2). Analysis of cortical thickness revealed no effect of group or group‐by‐time interaction in any regions within the temporal cortex VOI or across the entire cortex.

### 3.6 BrainPAD

**FIGURE 3:**
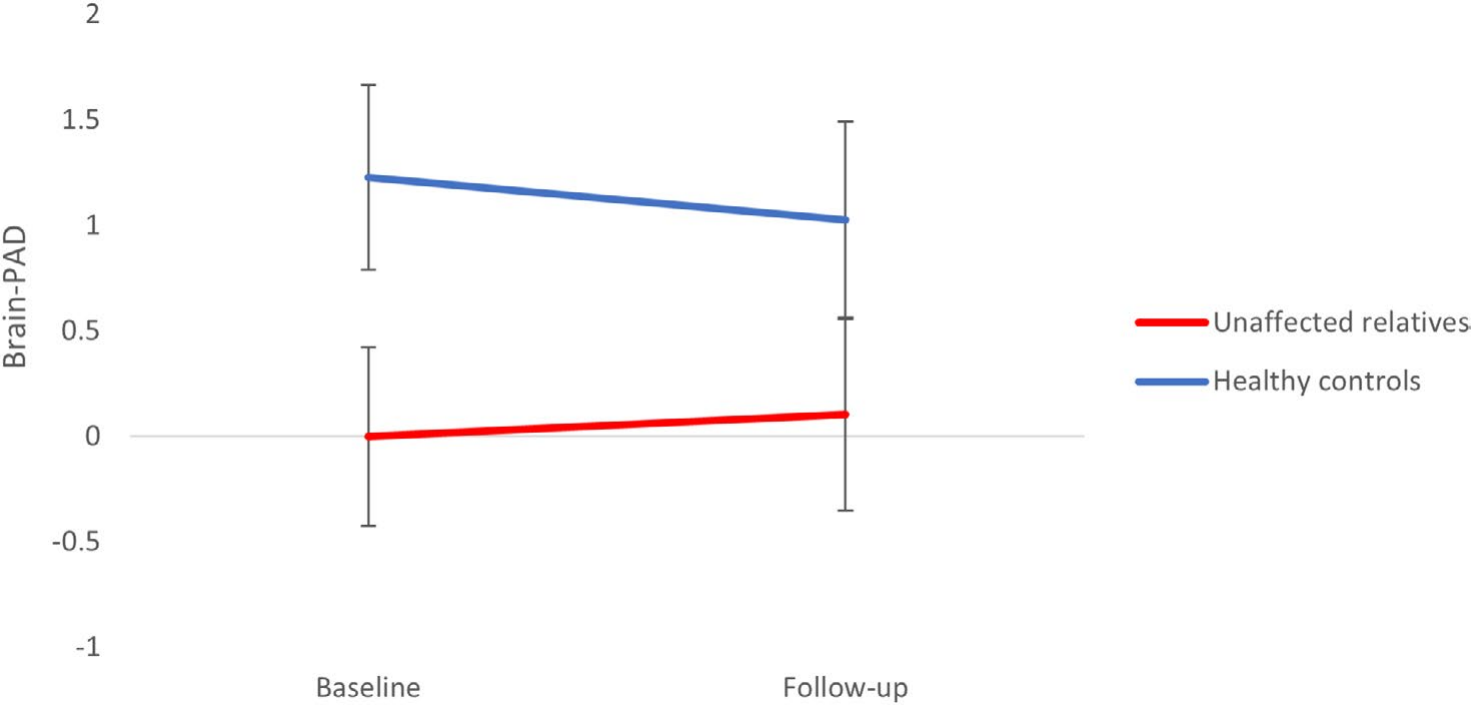
Non‐significant trend toward lower brain‐predicted age difference (brainPAD) in unaffected relatives (UR) compared to healthy controls (HC) across baseline and 15‐month follow‐up (*p* = 0.08). BrainPAD values are adjusted for age and sex, and error bars reflect standard error of the mean.

Results revealed a non‐significant trend toward a group difference in brainPAD (F (1, 107.73) = 3.21, *p* = 0.076), driven by UR generally showing lower brainPAD values across both timepoints compared to HC with no significant trajectory difference between groups (*p* = 0.30) (age‐ and sex‐adjusted means and standard errors at baseline: UR −0.04 (0.42), HC +1.23 (0.44); and follow‐up: UR +0.10 (0.45), HC +1.03 (0.46)) (Figure 3).

### 3.7 Post Hoc Correlation Analyses

Larger GM volume in the STG was associated with more subsyndromal mania symptoms in UR (*r* = 0.31, *p* = 0.009). This significant *p*‐value was, however, reduced to a non‐significant trend after adjusting for multiple comparisons (FDR adjusted *p* = 0.08). There were no significant associations between aberrant structure, functioning, or depressive symptoms (FDR adjusted ps ≥ 0.21).

## 4 Discussion

In this longitudinal study, we followed the evolution in neurocognitive functioning, brain structure, and brainPAD in UR of patients with BD over a 15‐month follow‐up period. UR showed initial subtle impairments in processing speed at baseline (z = −0.3), which normalized over the follow‐up time. They also displayed initially larger GM volume in STG, which also normalized over time. Across both time points, UR showed greater bilateral amygdala volume, as well as a trend toward lower brainPAD compared to HC. There were no differences between UR and HC in cortical thickness in the temporal cortex or across the entire cortex, or in total cerebral WM or hippocampal volume.

The amygdala is a key structure of the limbic system and is a particularly important node in the neural pathway involved in emotion processing and regulation [53]. Our observation of stably enlarged bilateral amygdala volume in UR at both baseline and follow‐up compared to HC was therefore interesting. Previous cross‐sectional studies provided inconsistent results [17]. Studies of young unaffected offspring 12–13 years of age [54, 55]; and adolescents/young adults 19–21 years of age [25, 56]; generally found normal amygdala volume. Enlarged amygdala volume was demonstrated in adult UR in the current and previous studies [57, 58]. This suggests a role of age on amygdala volume in at‐risk and BD populations [58, 59]. We previously found stably enlarged bilateral amygdala volume in remitted *newly diagnosed* BD patients over a 16‐month follow‐up period [60] whereas patients with more established illness, particularly BD type I, show *reduced* amygdala volume [61]. The larger amygdala volume seen in UR may thus reflect a vulnerability indicator, but with illness onset, there is progressive atrophy associated with illness progression.

The lack of significant difference between UR and HC in brainPAD is in line with the only two published studies of brainPAD in UR that found no baseline difference in offspring of parents with BD compared to HC [31, 32]. In fact, De Nooij et al., 2019 found that those UR who developed a mood disorder during the 2‐year follow‐up time presented the greatest decelerating trajectory over time compared to UR who remained well and HC [32]. Another few studies found that BD patients in the *early* stages of the disorder presented with younger (i.e., *lower*) brainPAD compared to HC [10, 31], whereas patients with more established illness exhibit significantly *larger* brainPAD compared to HC in a recent meta‐analysis [62]. The UR in the current study remained unaffected during the follow‐up time and showed a non‐significant trend toward *lower* brainPAD trajectory compared to HC. Evidence of a lower brainPAD in UR—and recently diagnosed patients—has been suggested to indicate a *delay* in normative age‐related brain structural changes during prodromal and early stages of illness, reflecting less synaptic pruning and deviation in brain maturation [10]. While the evidence is still sparse and conflicting, the slightly younger brain‐age estimation in UR may suggest that family predisposition to BD is associated with *some* abnormalities in neurodevelopment in a subgroup of UR at particularly high risk. Longer follow‐up period is needed to differentiate between UR who later develop psychiatric illness versus those who remain healthy.

This study is the first to demonstrate a significant trajectory difference between UR and HC in cortical surface in a region in the left STG, driven by UR displaying initially larger GM volume compared to HC, which normalized over time. The STG is important in facial emotion perception and social cognition [63] and prior functional neuroimaging studies in both BD and UR have found evidence of STG dysfunction during emotional processing, e.g., [64, 65, 66], and during rest, e.g., [67]. However, *prospective* studies of UR have been inconsistent—with one study showing accelerated volume reduction in right lateralized frontal (but not temporal) regions during a 2‐year follow‐up [22] and another showing stable greater GM volumes in several structures—including temporo‐parietal regions—in high‐risk versus low‐risk twins over a 7‐year follow‐up [24]. Differences between studies may be due to different samples of UR (mood disorders versus BD), follow‐up times, and statistical methodologies. In UR, larger STG volume and subtle processing speed impairments at baseline normalized over the follow‐up time. It is plausible that the greater STG volume and poorer processing speed in our sample of UR reflect a marker of risk that decreases with increasing age. However, more longitudinal studies with longer follow‐up periods are needed to clarify the association between STG volume, processing speed, and risk.

Strengths of the study include the longitudinal design with neurocognitive and neuroimaging data acquired at two timepoints and well‐defined moderate‐sized samples of first‐degree UR of patients with newly diagnosed BD and matched HC. Moreover, all participants underwent careful assessment of psychiatric illness, ensuring all participants had no personal lifetime history of mental disorders and were free of psychotropic medications, thereby enabling direct comparisons between the two groups to investigate endophenotypic markers associated with risk without the confounding effect of psychopathology and medication. The caveat to this is, however, that our UR sample may not be representative of high‐risk samples in general, as seen from their generally normal scores on FAST, reflecting a high overall functioning and mostly comprising siblings of BD patients. Moreover, the UR in the study had a mean age of 27; thus, the longitudinal period investigated mostly does not overlap with the period of vulnerability. Consequently, the present sample may be more resilient than other high‐risk populations that often exhibit prodromal symptoms (e.g., anxiety and attention deficit hyper‐activity disorder) that are more likely linked with larger familial disposition to psychiatric illness compared to lower familial load of psychiatric disorders in the present sample [68]. Thus, due to potential selection bias, the present findings could be interpreted as resilience markers. A limitation is the smaller number of participants at follow‐up (51% of original sample), which was due to limited financial resources preventing repeated MRI scanning of the entire sample, i.e., a factor unrelated to the population in question. Consistent with this, sensitivity analyses showed that the sample with assessments at both timepoints was comparable to the sample with only baseline assessment (Tables S1 and S2). Further, the proportion of the sample included in the follow‐up assessment is in line with previous longitudinal studies investigating structural change over time in UR versus HC that did not exclude participants with only one scan, e.g., 44%–71% of the original sample [23, 32, 69].

This longitudinal MRI study of UR of patients with BD versus HC over a 15‐month follow‐up period showed that UR had (i) stable enlargement of bilateral amygdala, reflecting a putative risk endophenotype for BD, (ii) no significant difference in brainPAD values compared to HC, and (iii) larger GM volume in STG and impairments in processing speed at baseline, reflecting possible markers of risk that decreased over the follow‐up time. Notably, our sample of UR remained unaffected during the short follow‐up time, and a majority of the UR had surpassed the peak period of vulnerability; thus, the UR sample may be more resilient than other high‐risk populations. Further research in at‐risk UR with longer follow‐up times is therefore needed to better understand the underlying neurobiological mechanisms of these findings and to elucidate whether structural abnormalities are predictive of risk or reflect markers of resilience.

## Conflicts of Interest

H.L.K. within the last 3 years received honoraria from Lundbeck. G.M.K. has the past 3 years received payment as speaker of Sage Therapeutics, H.L.K. and Angelini and has served as consultant for Sanos, Gilgamesh, Onsero and Pangea. S.S. has been sponsored by Janssen to attend a conference in the past 3 years. M.V. has received consultancy fees from Lundbeck and Janssen Cilag the past 3 years. L.V.K. has within the preceding 3 years been a consultant for Lundbeck and Teva. K.W.M. has received consultancy fees from Lundbeck and Janssen‐Cilag in the past 3 years. The remaining authors declare no conflicts of interest.

## Supporting information


**Data S1:** bdi70050‐sup‐0001‐DataS1.docx.

## Data Availability

The data that support the findings of this study are available upon reasonable request.
