# Supplementary material for: Longitudinal Trajectory of Cognition, Brain Morphometry, and Brain Predicted Age in Unaffected First‐Degree Relatives of Patients With Bipolar Disorder
Source: Bipolar Disord. 2025 Aug 13;27(6):424–34. doi: 10.1111/bdi.70050 (PMC12483307; doi:10.1111/bdi.70050)
Supplement: Supplementary file 1 — Data S1: bdi70050‐sup‐0001‐DataS1.docx. [file BDI-27-424-s001.docx]

**Supplementary material**

**Table S1.** Sensitivity analysis comparing baseline demographic and neurocognitive variables in unaffected relatives with baseline MRI only and unaffected relatives with MRI at both timepoints

|  | Baseline assessment only | Both assessments | P-value |
| --- | --- | --- | --- |
| N | 40 | 32 |  |
| **Demographic and clinical variables** |  |  |  |
| Sex, n (% female) | 20 (50%) | 16 (50%) | 1.00 |
| Age | 26.85 (7.37) | 28.06 (6.99) | 0.48 |
| Predicted full-scale IQ | 110.55 (5.68) | 110.01 (7.91) | 0.75 |
| Years of education | 14.95 (3.14) | 15.05 (2.61) | 0.89 |
| HDRS, total score | 1.69 (2.52) | 1.31 (1.77) | 0.47 |
| YMRS, total score | 0.51 (1.28) | 1.16 (1.80) | 0.09 |
| FAST, total score | 4.72 (8.18) | 2.97 (4.54) | 0.29 |
| Quality of life, EQ-5D index score | 0.95 (0.08) | 0.97 (0.06) | 0.33 |
| **Neurocognitive functioning** |  |  |  |
| Global cognition | -0.19 (0.48) | -0.07 (0.42) | 0.29 |
| Processing speed | -0.28 (0.69) | -0.22 (0.60) | 0.68 |
| Sustained attention | 0.06 (0.76) | 0.19 (0.75) | 0.46 |
| Verbal learning and memory | -0.28 (1.01) | -0.10 (0.95) | 0.44 |
| Working memory and executive functions | -0.25 (0.49) | -0.17 (0.49) | 0.50 |
| Abbreviations: HDRS=Hamilton Depression Rating Scale; YMRS=Young Mania Rating Scale; FAST=Functioning Assessment Short Test; EQ-5D=European Quality of Life - 5 Dimensions | | | |

**Table S2.** Sensitivity analysis comparing baseline demographic and neurocognitive variables in healthy controls with baseline MRI only and healthy controls with MRI at both timepoints

|  | Baseline assessment only | Both assessments | P-value |
| --- | --- | --- | --- |
| N | 27 | 38 |  |
| **Demographic and clinical variables** |  |  |  |
| Sex, n (% female) | 17 (63%) | 23 (61%) | 0.84 |
| Age | 29.00 (7.52) | 29.95 (10.96) | 0.70 |
| Predicted full-scale IQ | 112.39 (4.65) | 112.99 (4.89) | 0.63 |
| Years of education | 16.30 (2.14) | 15.32 (2.30) | 0.09 |
| HDRS, total score | 1.30 (1.20) | 0.82 (1.11) | 0.10 |
| YMRS, total score | 1.07 (2.24) | 0.55 (1.25) | 0.23 |
| FAST, total score | 1.37 (1.82) | 1.03 (1.57) | 0.42 |
| Quality of life, EQ-5D index score | 0.97 (0.06) | 0.98 (0.05) | 0.80 |
| **Neurocognitive functioning** |  |  |  |
| Global cognition | -0.07 (0.46) | 0.04 (0.62) | 0.43 |
| Processing speed | -0.02 (0.51) | -0.01 (0.85) | 0.96 |
| Sustained attention | -0.04 (0.78) | 0.02 (0.95) | 0.78 |
| Verbal learning and memory | -0.17 (0.87) | 0.12 (0.85) | 0.18 |
| Working memory and executive functions | -0.05 (0.60) | 0.04 (0.63) | 0.57 |
| Abbreviations: HDRS=Hamilton Depression Rating Scale; YMRS=Young Mania Rating Scale; FAST=Functioning Assessment Short Test; EQ-5D=European Quality of Life - 5 Dimensions | | | |
